# NDRG1 regulates Filopodia-induced Colorectal Cancer invasiveness via modulating CDC42 activity

**DOI:** 10.7150/ijbs.56694

**Published:** 2021-04-17

**Authors:** Batuer Aikemu, Yanfei Shao, Guang Yang, Junjun Ma, Sen Zhang, Xiao Yang, Hiju Hong, Galiya Yesseyeva, Ling Huang, Hongtao Jia, Chenxing Wang, Lu Zang, Jing Sun, Minhua Zheng

**Affiliations:** 1 Department of General Surgery, Ruijin Hospital, Shanghai Jiao Tong University School of Medicine, Shanghai, China.; 2 Shanghai Minimally Invasive Surgery Center, Ruijin Hospital, Shanghai Jiao Tong University School of Medicine, Shanghai, China.; 3 Department of Surgery, Shanghai Key Laboratory of Gastric Neoplasms, Shanghai Institute of Digestive Surgery, Ruijin Hospital, Shanghai Jiao Tong University School of Medicine, Shanghai, China.

**Keywords:** colorectal cancer, NDRG1, cytoskeleton, invasion, CDC42

## Abstract

N-myc downstream regulated gene-1 (NDRG1) has been identified as a putative metastasis suppressor gene and proved to be a key player in cancer spreading and proliferation in our previous work. However, the effects of NDRG1 on tumor invasion and the mechanisms behind it are rarely understood. Here we provided *in silico* evidence that NDRG1 plays a crucial role in actin reorganization in colorectal cancer (CRC). Through *in vitro* experiments, we next observed filopodia formation was altered in NDRG1-modified cell lines, while cell division cycle-42 (CDC42) displayed excessive activation in NDRG1-silenced cells. Mechanistically, NDRG1 loss disrupts the binding between RhoGDIα and CDC42 and triggers the activation of CDC42 and the downstream cascades PAK1/Cofilin, thereby promotes the formation of filopodia and invasiveness of CRC. The knockdown of NDRG1 led to enhanced dissemination of CRC cells *in vivo* and correlates with active CDC42 expression. Using clinical sample analysis, we found an elevated level of active CDC42 in patients with advanced T stage, and it was negatively related to NDRG1 expression. In sum, these results uncover a mechanism utilized by NDRG1 to regulate CDC42 activity in coordinating cytoskeleton reorganization, which was crucial in cancer invasion.

## Introduction

Colorectal cancer (CRC) is the third most commonly diagnosed cancer in both men and women worldwide 1, and its incidence and mortality rates in China over the past decades have been on an upward trend 2. In the early phase of metastatic spread, CRC cells rely much on the actin cytoskeleton's dynamic reorganization to change shape to gain mechanical support that promotes cell motility and invasion 3.

N-myc downstream regulated gene-1 (NDRG1), identified as a metastasis suppressor recently, has been proven to be a key player in affecting cancer proliferation, spreading, cell adhesion, and autophagy 4-6. Our team has recently reported new mechanisms of NDRG1 in regulating the proliferation of CRC cells 7 and inhibiting EMT through its interaction and promotion of caveolin-1 8 and found out its potential role in regulating stress fibers assembly 9. Despite the limited studies that displayed possible functions of NDRG1 during actin-filament assembly in prostate and oral squamous cell cancers 10, 11, no adequate explanation has been given for the underlying mechanism.

Actin-cytoskeleton reorganization regulates cell morphological changes, namely lamellipodia and filopodia formation, and results in the directional migration and invasion of cancer cells 12. As one of the critical factors in tumor metastasis, the activation of cytoskeletal proteins triggers the beginning of an invasive or metastatic process 13 and sometimes facilitates the survival of extravasated tumor cells 14. This migratory strategy of cancer cells to acquire optimum shape and obtain protrusive force is thought to be controlled by Rho GTPases, a family of small signaling G proteins 15, 16. Of the numerous functions Rho GTPases have shown in both normal and cancerous cells, the actin cytoskeleton's remodeling is one of the most remarkable. It could regulate the dynamics of cell morphology and motility by interacting with various downstream signaling molecules. For example, filopodium is an actin-rich protruding structure that extends from the plasma membrane in a fingerlike manner, and it has been thought to be largely influenced by the Rho GTPases activation 17. It serves as a hub where diverse cellular processes orchestrate and is closely involved in cell invasion and migration both *in vitro* and *in vivo* 17.

Notably, despite the preliminary evidence indicating an association between NDRG1 and actin reorganization, the precise mechanism of the metastasis suppressor in intervening cell protrusion remains unclear. We therefore investigated whether NDRG1 played a role in actin structure remodeling of CRC and, if so, what the functional role and clinical importance are. To the best of our knowledge, this work is the first attempt to demonstrate that the suppression of the CDC42/PAK1/Cofilin pathway, a key regulator of filopodia formation, underlies the ability of NDRG1 to inhibit CRC invasion. Mechanistically, NDRG1 could stabilize the RhoGDIα-CDC42 interaction and thereby affect the oscillation of CDC42 activation. Thus, the potential influence of NDRG1 in cytoskeleton reorganization via regulating actin filament depolymerization might give a better insight into the crucial role that NDRG1 plays in cancer invasiveness.

## Materials and Methods

### Cell culture and transfection

The HCT116 and RKO colon cancer cell lines were purchased from the American Type Culture Collection (ATCC, Manassas, VA). All cells were cultured in RPMI 1640-medium supplemented with 10% fetal bovine serum (FBS) and maintained at a 37 °C incubator with a 5% CO_2_ humidified atmosphere. The NDRG1 overexpression and knockdown clones were established as described previously 9. For transient transfection, cells were seeded in a 6-well culture plate 24 h before transfection; then cells were transfected with corresponding siRNA or vector, using Lipofectamine 2000 (Invitrogen), according to the manufacturer's instructions. siRNA duplexes used were as follows: siCDC42#1: sense: 5'-CCUGAAGGCUGUCAAGUAUTT-3', antisense: 5'-AUACUUGACAGCCUUCAGGTT-3'; siCDC42#2: sense: 5'-GCUUGUUGGGACUCAAAUUTT-3', antisense: 5'-AAUUUGAGUCCCAACAAGCTT-3'; siCDC42#3: sense: 5'-CCGCUGAGUUAUCCACAAATT-3', antisense: 5'-UUUGUGGAUAACUCAGCGGTT-3'.

### RNA isolation and quantitative real-time qRT-PCR analysis

RNA extraction and qRT-PCR analysis were performed as described previously 8. The primer sequences of all genes were listed as follows:

NDRG1 (5′-CTGCACCTGTTCATCAATGC-3′ and 5′-AGAGAAGTGACGCTGGAACC-3′); GAPDH (5′-TTCAACAGCAACTCCCACTCTT-3′ and 5′-TGGTCCAGGGTTTCTTACTCC-3′); FGD1 (5′- AGATATACAGCACCAAGGGTTC-3′ and 5′-CACTACATGGAGAAGGGTGG-3′); PLEKHG2 (5′-GAACTGTTTTCTGGGAGCAATC-3′ and 5′-TGGAAGTCTGTGAATGATACCC-3′); PREX1 (5′-GCAATATGTCACCCAGATCAAC-3′ and 5′-GTAGGAGTCGCGATAACTCATG-3′); TUBA (5′-CAAAGTCAAGACCTCGTCAAAG-3′ and 5′-GGATCTTGAGTTTCTGATTGGC-3′); PLEKHG4 (5′-TGTCCAGGAAATTCCAGTTACC-3′ and 5′-GACTGAGGAGCTTTCTACTGTG-3′); ITSN1 (5′-TGGAGAAGTTCAAGGTCAGA-3′ and 5′-TGTCAGCAGCTCAGACTCCA-3′); ITSN2 (5′-GCGCAGTCTCTGATTGATTTAG-3′ and 5′- GAAGAAGGGCATTTCTAGCTTG-3′); ECT2 (5′-CTAAAGATGCTGTGTCGACATG-3′ and 5′-TTGCTCTTGATGCTCTACTCAA-3′); PLEKHG1 (5′-CTCTGAAACACTCGCTGCCTCTG-3′ and 5′-AGCATCAAGCACCACATCATAGCC-3′); FGD4 (5′-TGGGCTATGTGGTGGATGAAATGC-3′ and 5′-GCCACTTCTGCTTCAGTTCCTCAC-3′); ARHGAP12 (5′-GAACAGGTCTTATTCGTGATGC-3′ and 5′-CAAATGAGCGTGTCCTATTCTG-3′); ARHGAP26 (5′- ACCAACAAATTCATCAAGGAGC-3′ and 5′-TCAATCATCCGTATCCGTTCAT-3′).

### Scanning electron microscopy (SEM)

Cells seeded on coverslips for 36 h were fixed in 2.5% glutaraldehyde for 2 h at room temperature, washed in PBS for three times, dehydrated with graded ethanol at 4 °C, soaked in 100% acetone for 20 min, and 100% isoamyl acetate for 15 min and propylene epoxide for 20 min at 45 °C. The coverslips were then vacuumed and spray-coated with metal foil and put under the Field Emission Scanning Electron Microscopy (SU8010, Hitachi, Japan) for observation.

### Immunofluorescence staining and evaluation

Cells seeded on coverslips for more than 24 h were fixed in 4% formaldehyde for 30 min at room temperature, then permeabilizated with 0.1% Triton X-100 for 30 min. After that, cells were treated with the primary antibody overnight at 4°C, followed by incubation with species-specific secondary antibody for two hours at room temperature. The coverslips were mounted using an antifade mounting solution containing 4,6-diamidino- 2-phenylindole (DAPI; P36935, Invitrogen) after three-time washing with phosphate-buffered saline (PBS). Actin was stained with Myo10 (Rabbit, HPA024223, Sigma-Aldrich) and Rhodamine Phalloidin (R415, Invitrogen) following the manufacturer's instructions. Confocal images were captured using ZEISS LSM 880 confocal microscope with Airyscan (Carl Zeiss, Germany) or Leica TCS SP8 confocal microscope (Leica, Switzerland), and fluorescence quantification was measured as described previously 9. Co-localization analysis was performed via Leica Application Suite X (LAS X).

### Immunoblotting, GST-pull down, and immunoprecipitation assay

Briefly, in the immunoblotting assay, lysates of cell samples were separated by SDS-PAGE gel, transferred to a PVDF membrane, blocked with 5% milk, and incubated with primary antibodies, then species-specific secondary antibodies. The primary antibodies included:

NDRG1 (Rabbit, Cat.ab124689, Abcam); GAPDH (Mouse, Cat. 60004-1-Ig, Proteintech); Myo10 (Rabbit, HPA024223, Sigma-Aldrich); RAC1 (Rabbit, Cat.ab155938, Abcam); CDC42 (Rabbit, Cat.ab187643, Abcam), CDC42 (Mouse, Cat.sc-8401, Santa Cruz); CDC42^GTP^ (Mouse, Cat.26905, Neweast Bio); Phospho-Cofilin (Thr423) (Rabbit, Cat.2601, Cell Signaling Technology); PAK1 (Rabbit, Cat.2602, Cell Signaling Technology); Phospho-Cofilin (Ser3) (Rabbit, Cat.3313T, Cell Signaling Technology); Cofilin (Rabbit, Cat.5175T, Cell Signaling Technology); N-WASP (Rabbit, Cat.4848, Cell Signaling Technology); Profilin-1 (Rabbit, Cat.3246, Cell Signaling Technology); ARP2 (Rabbit, ab47654, Abcam); ARP3 (Rabbit, Cat.4738, Cell Signaling Technology); PLEKHG2 (Rabbit, Cat.ab180156, Abcam); RhoGDIα (Rabbit, Cat.ab133248, Abcam), RhoGDIα (Rabbit, Cat.2564, Cell Signaling Technology).

GST-pull down assay to detect active CDC42 and RAC1 was carried out as the protocol of Active CDC42 Detection Kit (Cat.8819, Cell Signaling Technology) and Active RAC1 Detection Kit (Cat.8815, Cell Signaling Technology).

Immunoprecipitation was performed as described previously 7. Briefly, cell lysis containing 300 mg protein was incubated with the CDC42 antibody (Rabbit, Cat.ab187643, Abcam) overnight at 4 °C. 30 μl of A/G magnetic beads (Pierce Crosslink Magnetic IP/Co-IP Kit, Cat.88805) from Thermo Fisher Scientific (MA, USA) was then added into the mixture and incubated 4 h at 4 °C. The beads were washed, and a low pH elution buffer is used to dissociate bound antigen from the antibody-crosslinked beads. A neutralization buffer is applied to prevent precipitation of the isolated antigen. Resuspended in loading buffer and incubated over 90 °C for 10 min, the supernatant was then separated on a 12.5% Bis-Tris gel. Secondary antibody for IP detection (Veriblot, Cat.ab131366, Abcam) was used to detect the specific protein in the immunoblotting assay.

### Invasion assay

Invasion assay using Transwell chambers (8 μm for 24-well plate; Corning Costar, NY, USA) was performed as previously described 18, and the incubating time of different cell-type was as indicated in the results.

### Peritoneal metastatic xenograft model

Male BALB/c nude mice at the age of about four weeks (Charles River, Beijing, China) were housed at a specific pathogen-free environment and randomly divided into two groups (5 mice in each) (Research Center of Experimental Medicine, Shanghai Jiao Tong University School of Medicine Affiliated Ruijin Hospital). HCT116 sh-NDRG1 cells and the negative control cells were lentivirally transduced firefly luciferase fusion vector (GeneChem, Shanghai, China), and stably transfected cells were selected with 10 μg/ml puromycin. The colon cancer cells were trypsinized and resuspended in 100 μl PBS which contained 5×10^5^ cells and injected into the mice's abdomen. Before the euthanasia, tumor distribution and mass were assessed by bioluminescence imaging (Caliper Life Sciences, USA) in the fourth week. The sample size was calculated with Spectrum living image software, and subsequently, peritoneal foci were checked by gross specimens and microscopy. All experiments adhered to the NIH Guide for the Care and Use of Laboratory Animals.

### Clinical patient tissue microarray and IHC analysis

The microarrays, which contained 86 colorectal cancer and paired adjacent normal paraffin-embedded specimens, were supplied by the Shanghai Minimally Invasive Surgery Center of Ruijin Hospital (Shanghai, China) following the guidelines by the Ethics Committee of Ruijin Hospital. Written and informed consent of all the cases was signed before the study. After immunohistochemical assay was conducted as described 18, two independent pathologists analyzed the expression levels of target proteins. The overall score was determined by multiplying the cellular staining proportion (0 = 0%, 1 = ≤ 25%, 2 = 26 to 50%, 3 = 51 to 75%, 4 = 76 to 100% positive) and intensity (0 = no staining, 1 = weak, 2 = moderate, 3 = strong). All cases were delimited into two equivalent subgroups by the median score of CDC42^GTP^. NDRG1 expression was considered high if the overall score was greater than six and low if it was six or less.

### Statistical analysis

Experimental data are presented as the mean ± S.D.; two-tailed Student's t-tests with unequal variance were performed using GraphPad Prism 8.0 for macOS (GraphPad Software). The chi-square test or Fisher's exact test was used for categorical variable comparison by IBM SPSS 22.0 for macOS (SPSS INC.). The TCGA dataset results were carried out by using the package in R version (http://www.r-project.org). Differences were considered statistically significant when P values were less than 0.05.

## Results

### NDRG1 loss results in increased filopodia formation and invasiveness of CRC cell lines

We previously demonstrated a novel thiosemicarbazone iron chelator regulating actin-filament reorganization via a mechanism involving NDRG1 9. To further clarify the functional role of NDRG1 in CRC, we first compared the NDRG1-low-expression (lower half) colorectal cancer samples with the NDRG1-high-expression (higher half) samples *in silico* using The Cancer Genome Atlas (TCGA). We extracted 1786 differential expressed genes (DEGs) with criteria of P<0.05 and |log^FC^|>1 (Figure S1A). Gene Ontology (GO) and Kyoto Encyclopedia of Genes and Genomes (KEGG) enrichment analysis revealed multiple DEGs involved in focal adhesion, extracellular matrix (ECM) receptor interaction, regulation of actin cytoskeleton, and binding of integrin and actin (Figure S1B-C).

**Figure 1:**
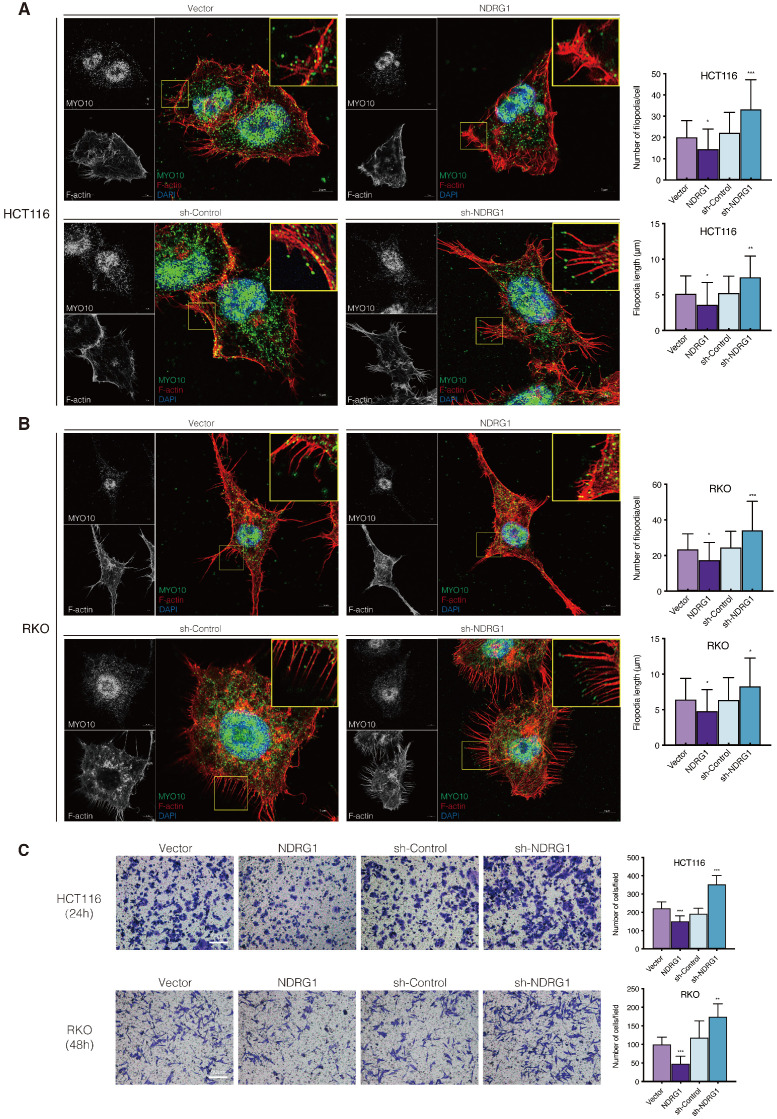
** NDRG1 loss results in increased filopodia formation and invasiveness in CRC cells. A-B)** Confocal images of the immunofluorescence staining of MYO10 (green) and rhodamine-phalloidin (red) accompanied by the cell nucleus (blue) in HCT116 (A) and RKO (B) cells. The insets show magnifications of MYO10-associated filopodial protrusions in the boxed areas. Quantification of the filopodial protrusions density and filopodia length is represented as mean ± S.D.; n>50 cells from three biological repeats; *P value <0.05, **P value <0.01, ***P value <0.001, relative to the respective control cells. Scale bar: 5 µm. **C)** Transwell invasion assay of HCT116 and RKO cells with NDRG1 overexpressing or silencing after incubating for 24 h (HCT116 cells) or 48 h (RKO cells). Data represent the mean ± S.D. of 3-5 different experiments. **P value<0.01, ***P value <0.001, relative to the respective control cells. Scale bar: 50 µm.

To obtain a broader understanding of NDRG1's role in actin remodeling and cell aggressiveness, we first established HCT116 and RKO CRC cell lines constitutively and stably express or knockdown NDRG1 (Figure S2A-B, P<0.001). With different traits in their cell phenotype and aggressiveness 19, these two cell models were introduced to show the consistency of different cell lines' responses. Next, we applied the immunofluorescence staining to visualize the cytoskeleton morphology. Co-stained with filopodia-specific binder Myosin-10 (Myo10), the structure of F-actin showed a remarkable alteration in the filopodial protrusion in the NDRG1-modified CRC cells. We observed clear, thin, and elongated protrusions outside the plasma membrane of both HCT116 and RKO cells, which matched the definition of filopodia 20 well. However, the plasma membrane protrusions were significantly decreased in number and weaker and shorter in size in NDRG1-overexpressed cells than vector cells (Figure 1A-B). Moreover, compared with the relative sparser and shorter filopodia presented in control cells, the F-actin filaments were aggregated into thick, abundant, and elongated membrane protrusions in the plasma and generated polarity in sh-NDRG1 cells of both cell lines (Figure 1A-B). We next applied scanning electron microscopy and observed that NDRG1-knockdown cells exhibited more elongated and enlarged filopodia, whereas relatively shorter and fewer microspikes presented in NDRG1-overexpression cells compared with respective control cells (Figure S3). These results collectively indicate that less NDRG1 might facilitate cytoskeleton rearrangement in colorectal cancer cells by promoting filopodia formation.

It is believed that during the event of invasive cancer cells penetrating the underlying stroma, filopodia play the role of pathfinder, which could degrade the basement membrane and then guide the cell body entering the compartment 21. We thereby observed the invasiveness of the CRC cells through matrigel-coated transwells and found that NDRG1-overexpressing showed an inhibitory effect on cell invasion while silencing NDRG1 distinctly upregulated cell invasion (Figure 1C, P<0.001 or P<0.01). This phenomenon was consistent in both cell types and could reflect the previous findings of morphological alteration on cancer cells.

### NDRG1 negatively regulates filopodia formation by inhibiting CDC42 activation

**Figure 2:**
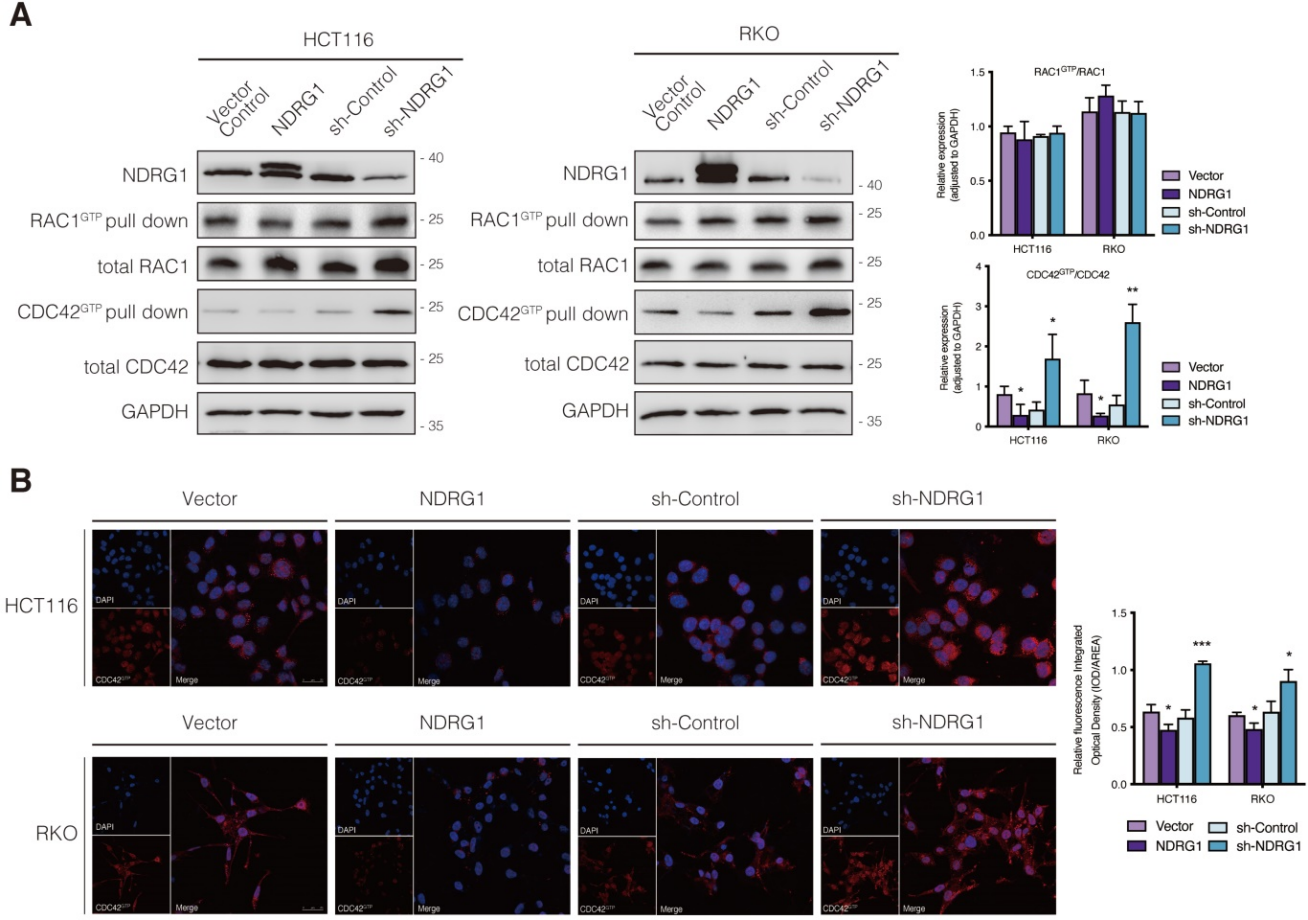
** Regulation of CDC42 activity by NDRG1 in CRC cells. A)** Immunoblotting for total protein level or activated form of indicated Rho GTPase in NDRG1-modified HCT116 and RKO cells. Results are representative of at least three biological repeats, and the values in histograms are represented by mean ± S.D.; *P value <0.05, **P value <0.01, relative to the respective control cells. **B)** Confocal images were taken to show immunofluorescence staining of active-CDC42 (red) accompanied by the cell nucleus (blue) stained by DAPI in NDRG1 overexpression and NDRG1 knockdown HCT116 and RKO cells relative to the control cells, respectively. Fluorescence quantification was performed by comparing the integrated optical density (IOD)/area value of active-CDC42 to the IOD/area value of the nucleus (DAPI) in the same image. Results are representative of three to five images from different visual fields, and the histogram values are mean ±S.D. *P value <0.05, ***P<0.001, relative to the respective control cells. Scale bars: 25 µm.

The small Rho GTPases are essential regulators of actin cycling and cytoskeleton reorganization. Activation of CDC42 has been proven to trigger filopodia formation and forward movement of motile cells, whereas RAC1 activity drives the extension of lamellipodia, which are broad and flat membrane protrusions always show up at the leading edge 12, 13, 22. They cycle between two conformational states, namely active state (bounding to GTP) and inactive state (bounding to GDP), and regulate many aspects of cancerous behavior 23. To further investigate whether the filopodia diversity observed in NDRG1-modified CRC cells was realized through the regulation of Rho GTPase family members, we detected the expression of the two major cell protrusion regulators; however, no significant alteration of the two proteins' expression was observed in either cell line (Figure 2A). Since the vital signaling function of RAC1 and CDC42 in cytoskeletal reorganization intensively depends on their biological activities, we next evaluated the transformation of active RAC1 and CDC42. Detection of RAC1 demonstrated no significant change, whereas CDC42 activation (*i.e.,* CDC42^GTP^) in both cell lines was dramatically (P<0.05) inhibited by over-expressed NDRG1 and markedly (P<0.01 or P<0.05) enhanced when NDRG1 was silenced (Figure 2A). These indicated that NDRG1 might reduce the activity of CDC42, to regulate actin cytoskeletal dynamics in colorectal cancer cells. In support of this hypothesis, we further performed immunofluorescence experiments to confirm the alteration of CDC42^GTP^ in both cell lines. The fluorescence intensity of CDC42^GTP^ was decreased (P<0.01 or P<0.05) and increased (P<0.001) in response to NDRG1 overexpression and knockdown, respectively (Figure 2B). These accumulative data suggest that NDRG1 suppresses the filopodia formation by diminishing the activity of CDC42.

### NDRG1-mediated filopodia rearrangement is regulated through a PAK1/Cofilin dependent pathway

To elucidate the cell invading mechanism triggered by over-activation of CDC42^GTP^, we next examined the downstream axis that targets actin filament reorganizing. Among the signaling pathways implicated in the CDC42-mediated filopodia formation, Wiskott-Aldrich syndrome protein (WASP) and insulin receptor tyrosine kinase substrate of 53 kDa (IRSp53) are canonical downstream targets, which could be activated by CDC42 and promote actin nucleation and polymerization by interacting with actin-related protein 2/3 (ARP2/3) complex^24^. Therefore, we asked through which pathway CDC42 impacted the cytoskeleton remodeling in an NDRG1-mediated manner. However, examining the protein level of the candidates above showed no significant change (Figure S4). Given that the ARP2/3-targeted signaling mainly contributes to the actin polymerization, we supposed that the cytoskeleton reorganizing might have been mediated via the process of actin dissembling.

**Figure 3:**
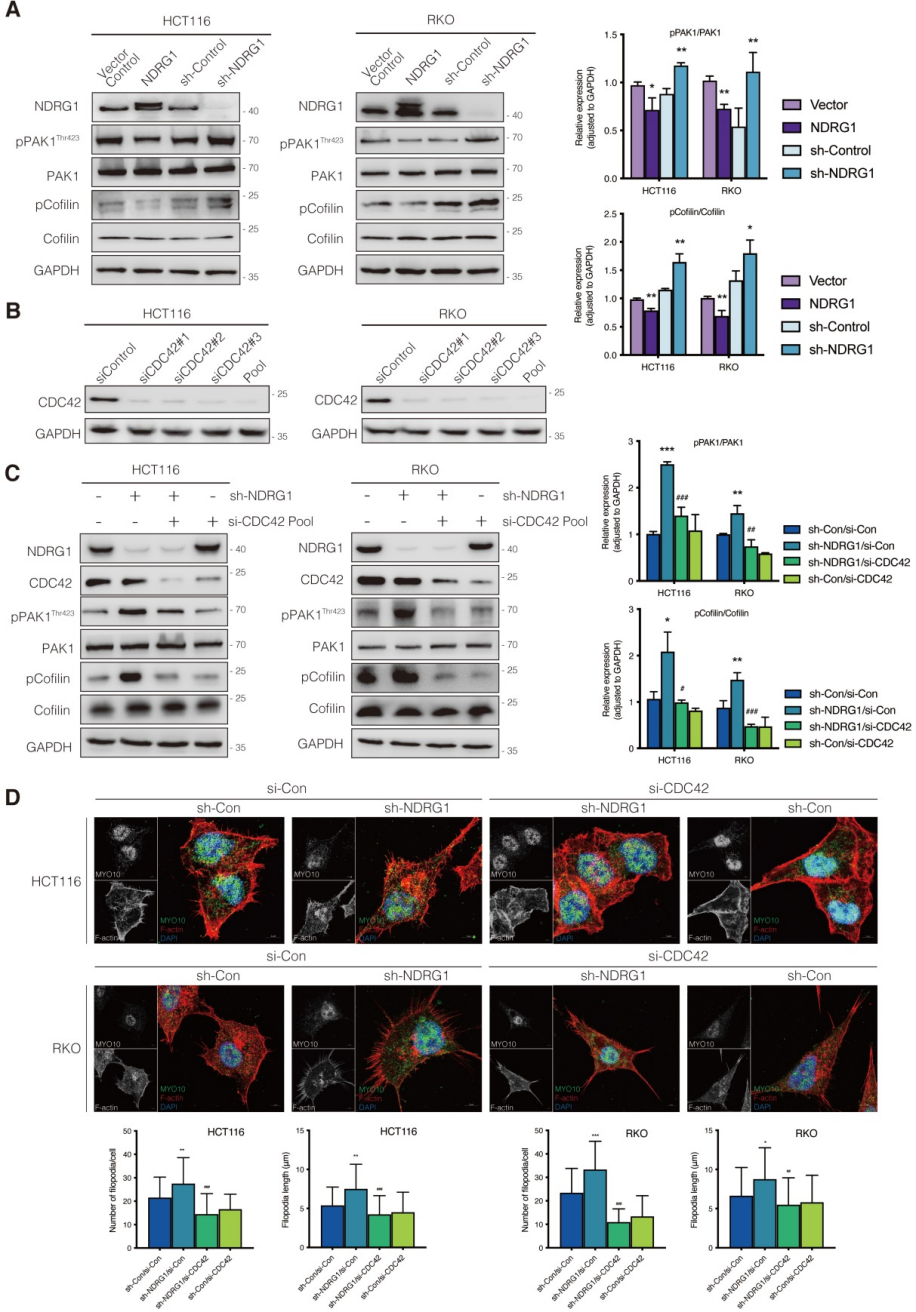
** Inhibition of CDC42 prevents NDRG1 loss induced CRC cell filopodial protrusion formation through suppression of PAK1/Cofilin signaling. A)** Immunoblotting analysis of the expression level of the total and phosphorylation form of PAK1 and Cofilin in indicated cell lines. **B)** Knockdown of CDC42 in HCT116 (left) and RKO (right) cells confirmed with immunoblotting analysis. Pool, combined siCDC42 sequences. **C)** Expression level of the total and phosphorylation form of PAK1 and Cofilin in indicated cell lines. **D)** Confocal images were taken to show immunofluorescence staining of MYO10 (green) and rhodamine-phalloidin (red) accompanied by the cell nucleus (blue) in colorectal cancer cells. Quantification of the MYO10-associated filopodial protrusions density and length is represented as mean ± S.D.; results are representative of 3-5 images from different visual fields, n>50 cells. *P value <0.05, **P value <0.01, ***P < 0.001, relative to the sh-Con/si-Con groups. ^#^P value <0.05, ^##^P value <0.01, ^###^P < 0.001, relative to the sh-NDRG1/si-Con groups.

Phosphorylation of p21-activated kinase 1 (PAK1), another downstream target for CDC42, could inhibit Cofilin's function and thereby triggers the debranching of actin filaments 25. To explore whether depolymerization was the reason for filopodial response upon NDRG1/CDC42 signaling, we next examined the activity of related markers involved. As shown in Figure 3A, total PAK1 or Cofilin expression was not affected when NDRG1 was overexpressed or knocked down in either HCT116 or RKO cells. Nonetheless, overexpression of NDRG1 significantly (P<0.01 or P<0.05) decreased the phosphorylation levels of PAK1 (*i.e.*, pPAK1/PAK1 ratio) and Cofilin (*i.e.*, pCofilin/Cofilin ratio), while knockdown of endogenous NDRG1 led to a significant (P<0.01 or P<0.05) enhancement of the phosphorylated form of PAK1 and Cofilin in both cell types (Figure 3A). The binding of RAC1/CDC42 to the PBD of p21-activated kinase (PAK) causes autophosphorylation and conformational changes in PAK, and it is known that phosphorylation of PAK1 at threonine 423 results in an increased PAK1 activity 26. PAK1 plays an essential role in cytoskeletal rearrangements by activating LIM kinase, which subsequently phosphorylates Cofilin into an inactive form and thus decreases the depolymerization rates 27.

To uncover the role of CDC42 in the NDRG1-mediated actin depolymerization signaling pathway, we next inhibited the cellular CDC42 process with the combined siRNA (Figure 3B). As expected, suppressing CDC42 in either NDRG1 knockdown or control cells could dramatically (P<0.001, P<0.01, or P<0.05) down-regulate the phosphorylation levels of PAK1 (Figure 3C) and reverse the NDRG1-silencing-induced pCofilin accumulation. Cofilin, a modulator that promotes the F-actin's debranching and severing, orchestrates cytoskeleton reorganization only in the non-phosphorylated form 28. These results confirm that CDC42 was the key mediator in the NDRG1-inhibited PAK/Cofilin signaling and cytoskeleton reorganization. Given that the role of PAK1/Cofilin in reforming filopodia was well characterized^29^, we further assessed the reversal effect of depleting CDC42 in cell protrusion formation by measuring filopodial density and length by rhodamine-phalloidin and Myo10 co-staining (Figure 3D). The quantification analysis corroborated our previous finding that loss of NDRG1 induced more aggressive growth of filopodia than in the control group. Moreover, the inhibition of CDC42 in sh-NDRG1/si-CDC42 groups of both cell lines reversed the overgrowth dramatically (Figure 3D, P<0.001) compared with sh-NDRG1/si-Con groups. Moreover, the over-invasiveness of CRC cells induced by silencing NDRG1 was impeded when inhibiting CDC42 (Figure S5, P<0.001). In sum, these results explain the phenotypic change in cancer cells and indicate an inhibitory role of NDRG1 in actin reorganization and cell invasiveness via a CDC42/PAK1/Cofilin dependent pathway.

### NDRG1 inhibits CDC42 activation by stabilizing the RhoGDIα-CDC42 binding

**Figure 4:**
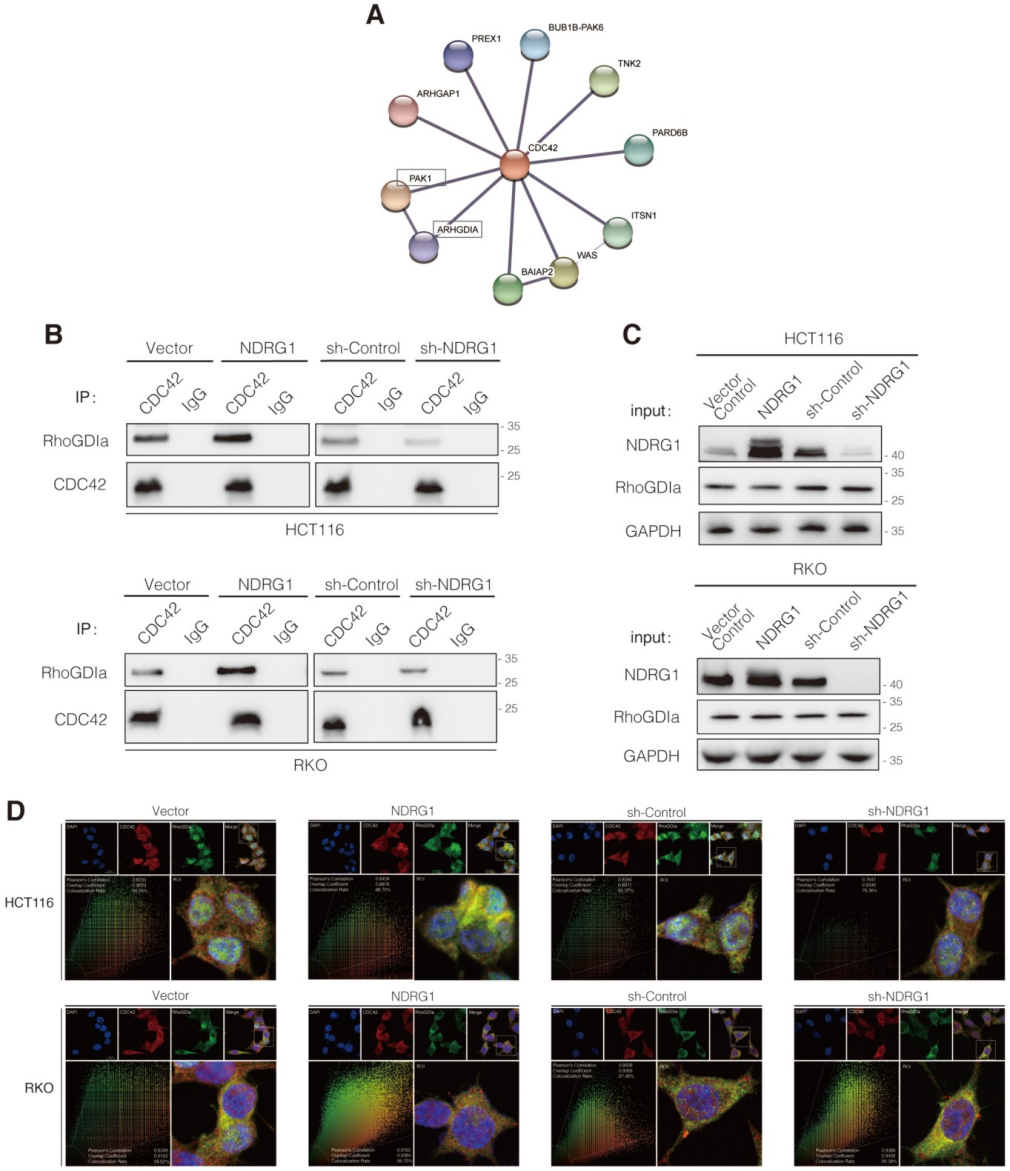
** NDRG1 suppresses CDC42 activity by stabilizing the RhoGDIα-CDC42 binding. A)** The STRING network view of interactive proteins of CDC42 in humans. Gray lines between the nodes indicate various types of interaction evidence. **B)** Co-immunoprecipitation to examine the interaction of RhoGDIα and CDC42 in both HCT116 and RKO cell lines. **C)** Immunoblotting assay to evaluate the influence of NDRG1 modification on RhoGDIα expression in indicated cells. GAPDH was used as loading control. **D)** Double stained confocal immunofluorescence assay and co-localization analysis to confirm the interaction of RhoGDIα and CDC42 in indicated cells (red: CDC42, green: RhoGDIα, blue: DAPI, scale bar: 20 µm). Co-localization analysis on wide-field merged images was performed via Leica Application Suite X. Results are representative of five images from different visual fields.

Our study has revealed the CDC42 over-activation after NDRG1-depletion and consequently upregulated filopodia formation (Figure 3); however, the mediators between NDRG1 and the Rho GTPase were still obscure. To find out the previously known or predicted interactors of CDC42 that possibly modified the NDRG1-regulated cytoskeleton reorganization, we next referred to the STRING database (http://string-db.org/) to depict the protein-protein interaction network of CDC42 in human organisms. Among the top ten interactors, there are members of guanine nucleotide exchange factors (GEFs), GTPase-activating proteins (GAPs), and Rho GTPase dissociation inhibitors (RhoGDIs) families, which are well-recognized Rho GTPases modulators (Figure 4A). We first detected the mRNA expression levels of the canonical modulators in the NDRG1-overexpressed and -silenced CRC cells (Figure S6A). Despite the partial increase of PLEKHG2 shown in NDRG1-knockdown HCT116 cells, which was not consistent with the further immunoblotting examining (Figure S6B), we observed no remarkable changes in other potent GEFs or GAPs of CDC42. We then hypothesized the effect of the activation might have been occurring through the binding of RhoGDIs with CDC42, which leads to the stabilization of the inactive form of CDC42 30.

To evaluate the binding of RhoGDIα and CDC42, co-immunoprecipitation (co-IP) was conducted. As shown, the binding was enhanced as NDRG1 was overexpressed; conversely, it decreased as NDRG1 was knockdown (Figure 4B) while the CDC42 quantity was consistent. Furthermore, the levels of RhoGDIα showed no significant difference in NDRG1-modified cells compared with the control cells (Figure 4C). We also detected the binding of NDRG1 to RhoGDIα and CDC42, but neither direct interaction was found through co-IP assay (data not shown). Further immunofluorescence staining confirmed that RhoGDIα has strong co-localization with CDC42 in both cell lines (Figure 4D). It has been previously confirmed that RhoGDIα is a key negative regulator of CDC42^GTP^ 31, 32. Our data suggest that, by stabilizing the complex of RhoGDIα and CDC42, NDRG1 plays a crucial role in keeping CDC42 in the inactive form and thereby prevents filopodia formation and cancer cell invasion. Once NDRG1 was lost, the cancer cells will develop into a more aggressive form with an excessively activated cytoskeleton-reorganization signaling axis.

### NDRG1-knockdown facilitates peritoneal dissemination of CRC cells and correlates with active CDC42 expression *in vivo*

**Figure 5:**
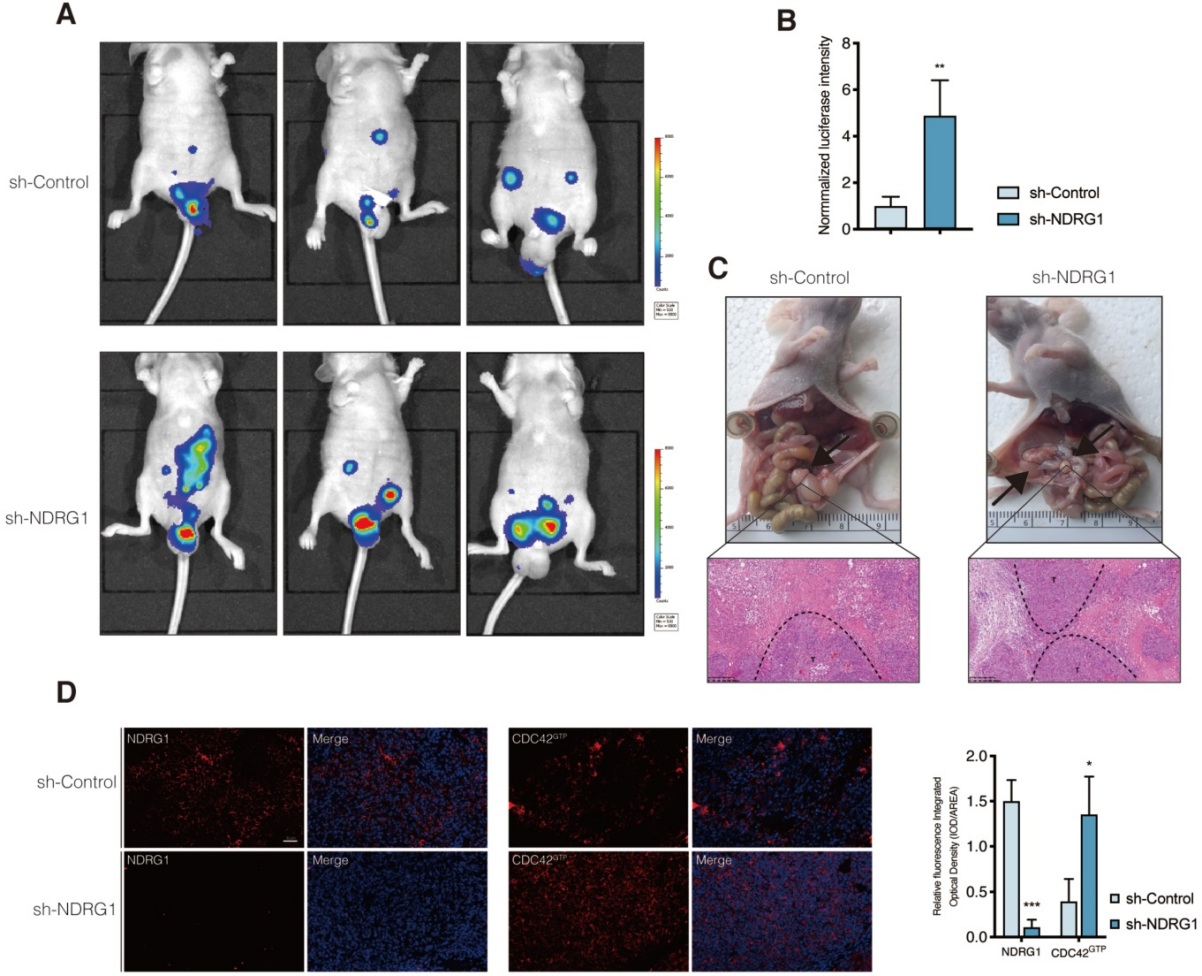
** Silence of NDRG1 promotes the peritoneal metastasis and correlates with upregulated CDC42^GTP^ expression. A)** Peritoneal metastasis of CRC cells in BALB/c nude mice. Tumors in two groups were measured *in situ* and assessed by bioluminescence imaging in the fourth week. **B)** Statistical analysis of the bioluminescence in peritoneal foci of both groups. Results are shown as mean ± S.D. **C)** Tumors in two groups are demonstrated after laparotomy with hematoxylin-eosin staining of peritoneal foci on the lower panel. Scale bars are as indicated. **D)** Immunofluorescence staining of NDRG1 (left) or CDC42^GTP^ (right) accompanied by the cell nucleus stained by DAPI in peritoneal foci derived from sh-NDRG1 and control groups. Results are representative of 3-5 images from different visual fields and the histogram values are mean ± S.D.; *P value <0.05, ***P< 0.001, relative to the respective control groups. Scale bar: 50 µm.

Given that the filopodium-like protrusions enable the outgrowth of cancer macrometastases at distal sites 33, we asked whether the depletion of NDRG1 could promote the peritoneal tumor macrometastases of colorectal cancer *in vivo*. We first engineered luciferase-green fluorescent protein (GFP)-tagged sh-NDRG1 and sh-Control HCT116 cells and injected them intraperitoneally into nude mice. Not surprisingly, the bioluminescent imaging showed the tumor burdens of the sh-NDRG1 group were more massive than that of controls in the peritoneum of hosts (Figure 5A-B, P<0.01). Immunofluorescence staining of NDRG1 and CDC42^GTP^ was performed to examine further their correlation in disseminated peritoneal foci, which was first confirmed as CRC metastases by pathology (Figure 5C). The results demonstrated a higher expression of CDC42^GTP^ in the NDRG1-knockdown group than the control (Figure 5D, P<0.05). The above findings indicated that silencing NDRG1 facilitates peritoneal dissemination of colorectal cancer, and it is correlated with an elevated level of active CDC42.

### Active CDC42 was frequently upregulated in CRC and was negatively related to NDRG1 expression

**Figure 6:**
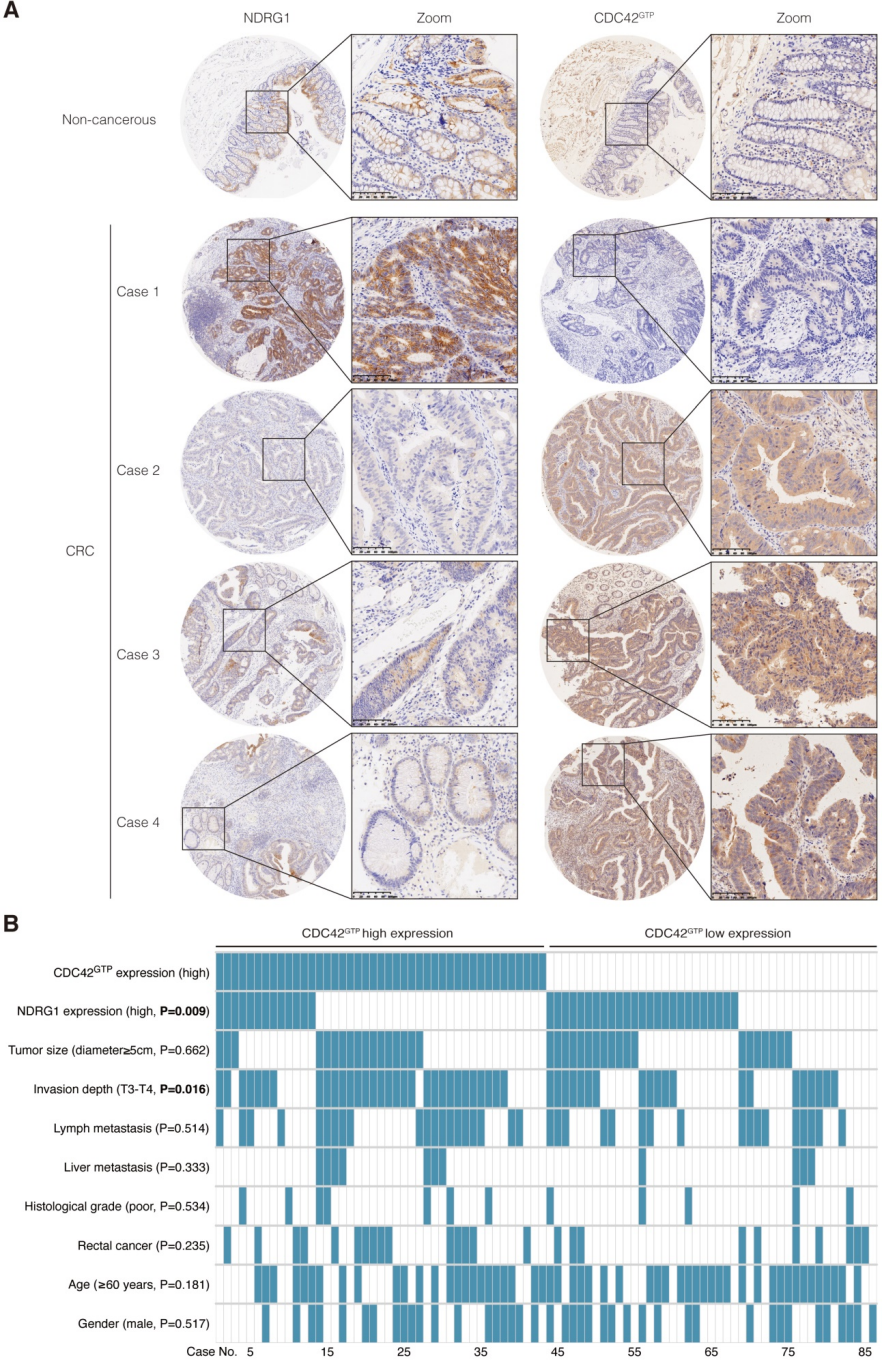
** CDC42^GTP^ is frequently upregulated in CRC tissues and correlated with NDRG1 expression and clinicopathological parameters. A)** IHC staining of NDRG1 and active CDC42 expression in tumor and adjacent tissues in microarray. Magnification on the right with a scale bar of 100 µm. **B)** Heatmap illustrating different clinicopathological parameters between CDC42^GTP^-high and -low-expression tumors of the 86 cases. Statistical significance was analyzed by the χ^2^ test. P values are as indicated.

In our previous study, NDRG1 was remarkably reduced in CRC tissues and was negatively correlated with tumor stage and metastasis 8. According to our extensive literature review, studies involving active CDC42 expression in human colon cancers were rarely reported. To gain further insight into the manner of NDRG1 and CDC42^GTP^ expression with clinicopathological parameters, we used tissue array containing 86 pairs of cancer and matched peritumor specimens of colorectal cancer patients. Consecutive slices from the same sample were applied in staining the two proteins (Figure 6A). We delimited the overall cohort of 86 patients into two groups by the median staining intensity of CDC42^GTP^ and discovered more robust CDC42^GTP^ expression in more advanced cancer (*e.i.*, T-stage was higher, Figure 6B, P=0.016). Moreover, high CDC42^GTP^ expression was associated with low NDRG1 expression, and vice versa (P=0.009). In the meantime, we assessed baseline information and other clinicopathological features, where the various intensity of CDC42^GTP^ made no significant difference (Figure 6B). These results collectively indicated that CDC42^GTP^ expression has a negative correlation with NDRG1 expression in CRC tissues and is associated with the advanced invasiveness of tumor.

## Discussion

**Figure 7:**
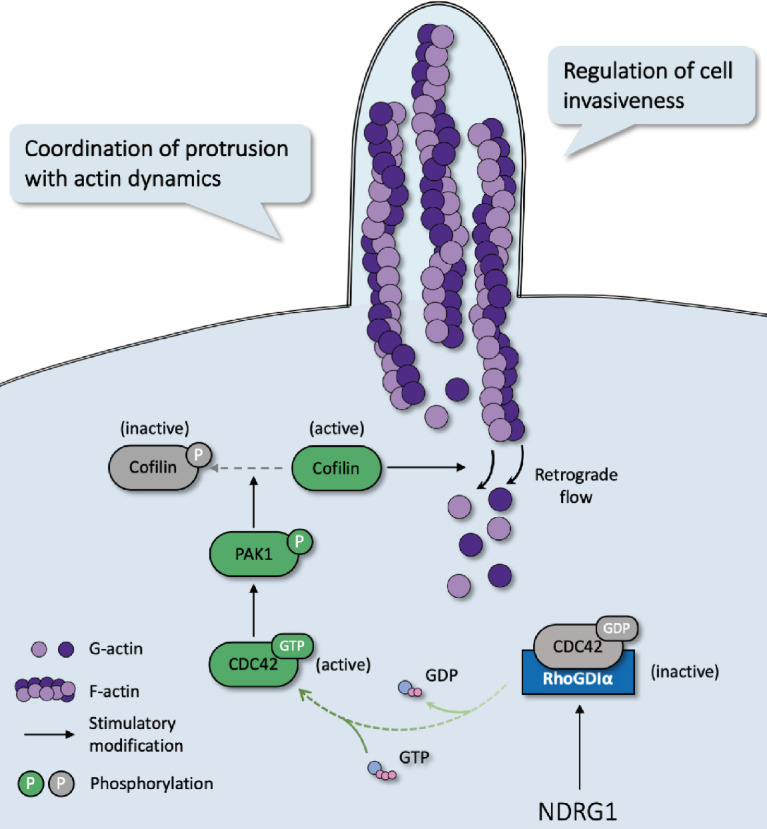
Schematic diagram for the mechanism of NDRG1's regulation of CDC42/PAK1/Cofilin axis as a switch that modulates actin cytoskeleton rearrangement in human colorectal cancer invasion by stabilizing the RhoGDIα-CDC42 binding.

In most malignancies, filopodia are associated with enhanced cancer cell invasion and metastasis and are a vital sign of actin-cytoskeleton reorganization. Based on our prior study that revealed the correlation of NDRG1 and stress fibers assembly 9 and the discovery of the potent mechanism of NDRG1 from public datasets (Figure S1), here we reported a novel mechanism of NDRG1 in regulating tumor invasion by mediating actin depolymerization in colorectal cancer. Using *in vitro*, *in vivo*, and tissue studies, we found that NDRG1 regulated actin cytoskeletal dynamics via modulating the activation of CDC42 and its downstream signaling pathway PAK1/Cofilin, which were realized by altering the stabilization of the RhoGDIα-CDC42 binding (Figure 7).

Filopodia, the actin-rich protruding projections that contribute to cell-cell communication 34, local invasion 35, directional migration 36, and cellular adhesions 37, are prominent features of invasive or immigrant cancer cells 38. Similar to other cellular protrusions like podosomes which were reported to promote cancer invasiveness by degrading extracellular matrix 39, filopodia plays a profound role in tumor cell movement, orchestrates the mesenchymal cell motility with its close coordination of adhesion at the leading edge 40. This cell motility process occurs through two possible mechanisms: (1) actin polymerization against the cellular membrane at the leading edge provides force 3 and/or (2) actin filaments form stress fibers composed of contractile actomyosin structures 41, 42. Cofilin promotes debranching and depolymerization of actin and promotes severing of the filaments into short segments 28, while phosphorylated Cofilin, an inactive form of the same protein, stops the disassembly of actin filaments 43. Moreover, the PAKs family composed of 6 members is initially determined as protein kinases that function downstream of the Rho GTPases CDC42 and RAC 44. PAK1, which belongs to Group I PAKs, is most often associated with carcinogenesis, where the PAK1/LIMK1/Cofilin signaling is frequently involved 29, 45, 46. On account of their well-characterized roles in cancer, we investigated the mechanism of actin filament reorganization in colorectal cancer cells in an NDRG1-modified manner. We discovered that NDRG1 inhibits cell invasion via the effects on PAK1/Cofilin-regulated actin filament debranching (Figure 3). To the best of our knowledge, this work is the first to point out that NDRG1-modulated cytoskeleton rearrangement and invasion are obtained through regulating the CDC42 activation.

Persistent activation of Rho GTPase and the subsequently regulated genes that drive actin filament reorganization have been demonstrated to be pivotal for tumor progression and, therefore, negatively affect the prognosis of patients 47, 48. Thus, targeting Rho GTPase and inhibiting the bypass signaling depicts a promising strategy for cancer therapy 49, 50. Our previous study revealed that NDRG1 exerts the inhibitory role in cell migration via Rho kinase-mediated regulation of pMLC2 (phosphorylated myosin II light chain) 9. Although Rho and CDC42 control actin-myosin contractility and the filopodia formation, respectively, there is convergence between the two Rho GTPases' signaling. For example, MRCK (myotonic dystrophy kinase-related CDC42 binding kinase) and ROCK cooperate in the myosin-dependent cell motility 51, while CDC42 and RhoA could act antagonistically in regulating membrane metalloproteinase localization 52. Moreover, Rho-ROCK activity is required in the motility and bleb-like protrusion formation in rounded tumor cells, like A375m2 melanoma, but not elongated F-actin-rich protrusive movement of cells like BE colon carcinoma 53. El Sibai et al. also reported that the inhibition of ROCK leads to a switch between CDC42- and RAC1-dependant membrane protrusion in rat mammary adenocarcinoma cells 54. In the present study, the observation of switched CDC42-activation using GST-pull down assay, a widely approved approach to accurately assess the activation of the Rho GTPase, has demonstrated a close relationship between CDC42 and NDRG1 (Figure 2).

Next, we exhibited that the inhibition of actin depolymerization via phosphorylation of PAK1/Cofilin could be responsible for the NDRG1-regulated actin reorganization, which was further confirmed by abolishing CDC42 (Figure 3). The silencing of CDC42 blocked the phosphorylation of PAK1/Cofilin and further diminished the excessive stimulation of the filopodia formation motivated by NDRG1 knockdown. Importantly, this novel axis revealed in our study demonstrates a mechanism by which NDRG1 exerts the anti-invasion effects through remodeling the actin cytoskeleton in colorectal cancer. Further investigations on clinical tissue and peritoneal dissemination model of nude mice also indicated that CDC42^GTP^ expression has a negative correlation with NDRG1 expression in CRC tissues and is associated with the advanced invasiveness of the tumor (Figure 5-6).

The function of CDC42 and other Rho GTPase in tumorigenesis is thought to be positively related to cell surface receptors and interaction with GEFs, GAPs, and RhoGDIs 30, 55. RhoGDIα is the most common and best-explained member of the family; it is ubiquitously expressed and interacts with several Rho GTPase 30 and functions as a crucial molecule that switches the oscillation of Rho GTPase between inactive and active states. The contact of RhoGDIα with GDP prevents its detachment from the GTPase or, contrarily, inhibits the binding of GTP and maintains the inactive state of CDC42 55. Through the Co-IP assay, we examined and proved the interaction of RhoGDIα and CDC42 was largely influenced in NDRG1-modified cells (Figure 4). When the binding of RhoGDIα to CDC42 increases in NDRG1-overexpression cells, it impedes the inappropriate activation of CDC42 and thereby inhibits the downstream signaling axis that controls cell morphology and polarization. Changes in the levels of expression of RhoGDIs have been correlated with several types of cancer, and feedback loops are coordinating the expression of both the Rho GTPases and RhoGDIs 56, 57; however, little is known about how the expression and binding of RhoGDIα are regulated 30.

In summary, our current findings represent fundamental advances in the novel mechanism underlying NDRG1's anti-cancer character. Rho GTPase signaling is versatile and indispensable to cell adhesion and motility 58. It is thus an important limitation that we evaluated the role of the two major Rho family members, CDC42 and RAC, in filopodia regulation and cell invasiveness, the effect of Rho and other actin-related molecules remains to be determined. Given the complicated crosstalk of Rho family GTPases, further investigation into the role of these genes and their interactions with NDRG1 is needed, and feedback regulating CDC42 oscillations and spatial self-organization remains to be solved. Further studies have yet to be carried out across more cancer types and in larger cohorts as well as in therapeutic agents, such as a novel iron chelator we are currently researching, to develop a more promising strategy in cancer therapies.

## Supplementary Material

Supplementary figures.
