# Supplementary material for: NDRG1 regulates Filopodia-induced Colorectal Cancer invasiveness via modulating CDC42 activity
Source: Int J Biol Sci. 2021 Apr 17;17(7):1716–30. doi: 10.7150/ijbs.56694 (PMC8120473; doi:10.7150/ijbs.56694)
Supplement: Supplementary file 1 — Supplementary figures. [file ijbsv17p1716s1.pdf]

Supplementary Figures

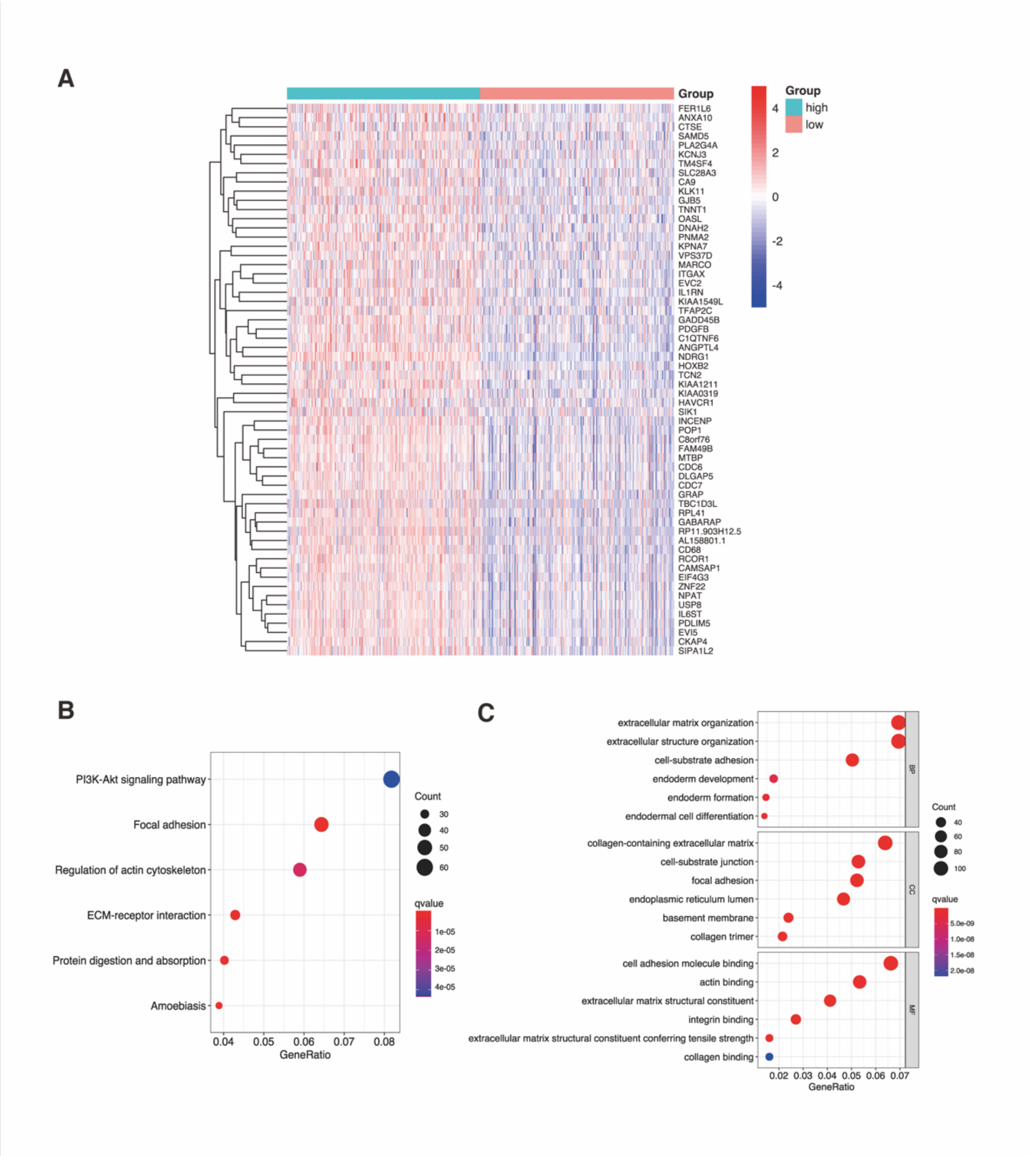

Figure S1. Association of NDRG1 and cytoskeleton regulation in CRC.

Based on the |log^{FC}|, the top and low 30 genes among the differential expressed genes (DEGs) extracted from NDRG1-low and -high-expression samples are exhibited by the heatmap (A). Gene Ontology (B) and Kyoto Encyclopedia of Genes and Genomes (C) enrichment analysis on DEGs. P values are as indicated. "limma" and "clusterProfiler" R packages were used for gene expression analysis and enrichment analysis, respectively.

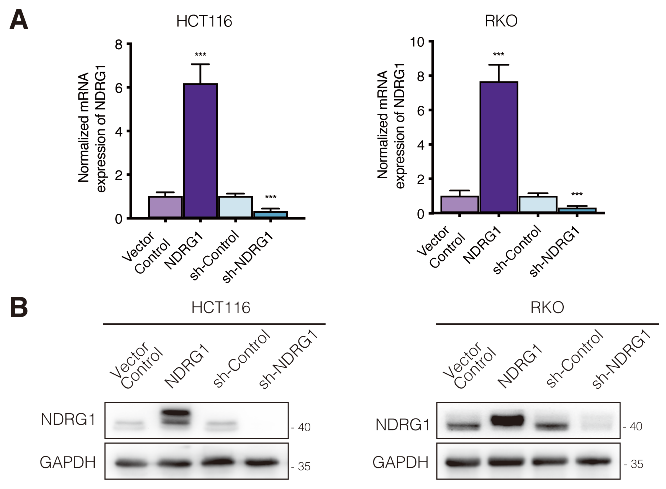

Figure S2. Establishment of NDRG1 over-expression and knockdown cell models.

qRT-PCR and immunoblotting results demonstrate NDRG1 over-expressing or loss at both the mRNA (A) and protein (B) levels in indicated cells. GAPDH was used as a loading control. ***P value<0.001, relative to the respective control cells.

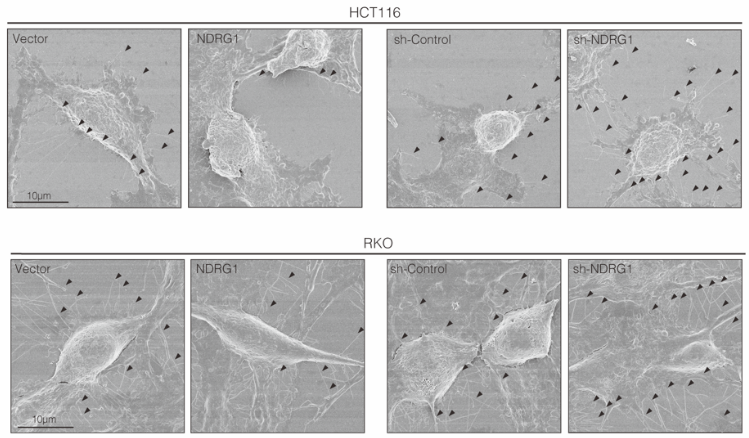

Figure S3. NDRG1 loss results in increased filopodia-formation of CRC cells.

Scanning Electron Microscopy (SEM) observation of cell morphology in HCT116 (A) and RKO (B) cells. Scale bar: 10µm. Black arrowheads denote representative filopodial protrusions.

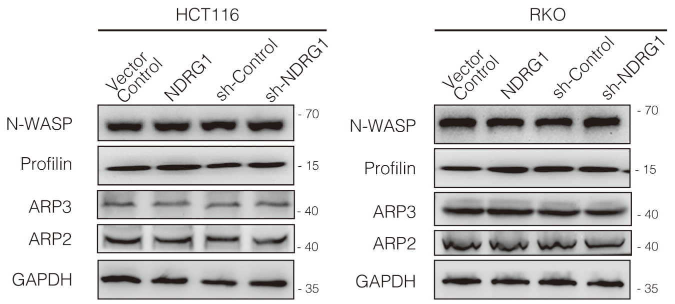

Figure S4. Immunoblotting analysis of the CDC42 downstream signaling pathway.

Representative immunoblotting analysis of the candidate proteins in the downstream of CDC42. GAPDH was used as loading control.

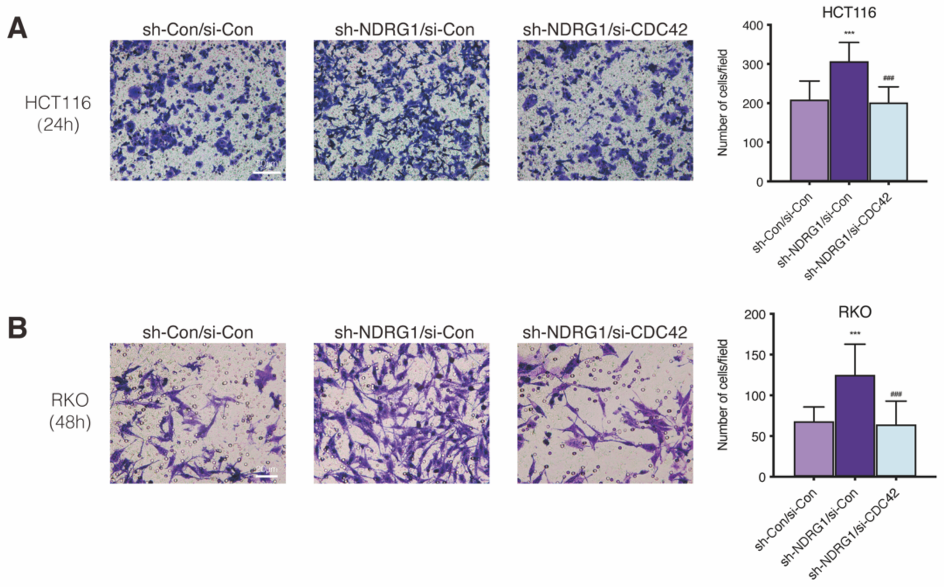

Figure S5. Inhibition of CDC42 impedes the sh-NDRG1-induced over invasiveness.

Transwell invasion assay of indicated HCT116 (A) and RKO (B) cells after incubating for 24h (HCT116 cells) or 48h (RKO cells). Data represent the mean ± S.D. of at least three biological repeats. ***P < 0.001, relative to the sh-Con/si-Con groups; ###P < 0.001, relative to the sh-NDRG1/si-Con groups. Scale bars are as indicated.

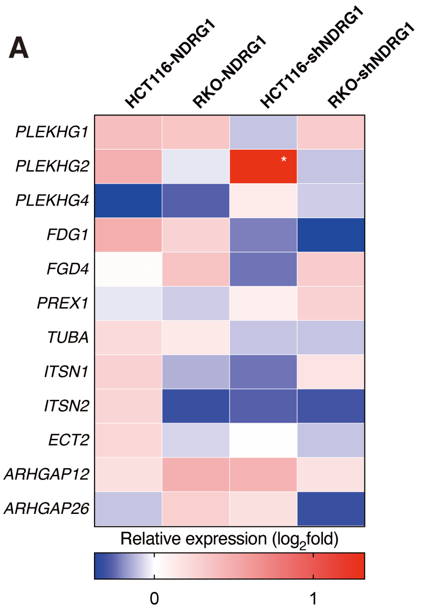

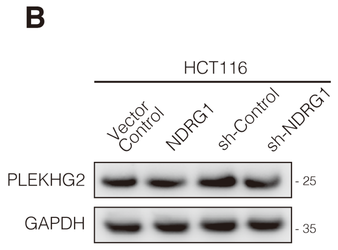

Figure S6. Effect of NDRG1 on CDC42-related GEFs and GAPs in transcription and protein expression level.

A) qRT-PCR analysis of potent GEFs and GAPs genes of CDC42 in indicated cells (normalized to GAPDH, adjusted to relative control groups, *P value<0.05).

B) Immunoblotting analysis of the expressions of PLEKHG2 in HCT116 cells. GAPDH was used as loading control. GEFs: guanine nucleotide exchange factors. GAPs: GTPase-activating proteins.
